# Expanding Access to Home-Based Behavioral Health Services for Children in Foster Care

**DOI:** 10.1007/s10488-024-01357-3

**Published:** 2024-04-02

**Authors:** Anna Chorniy, Michelle A. Moffa, Rebecca R. Seltzer

**Affiliations:** 1 Department of Medical Social Sciences, Feinberg School of Medicine, Northwestern University, Rubloff Building, 420 E. Superior St., Chicago, IL 60611 USA; 2 Buehler Center for Health Policy and Economics, Feinberg School of Medicine, Northwestern University, Chicago, IL USA; 3 Johns Hopkins School of Medicine, Baltimore, MD USA; 4 Johns Hopkins Bloomberg School of Public Health, Baltimore, MD USA

**Keywords:** Foster care, Pediatric mental health, Home-based behavioral health, Medicaid coverage

By age five, Rosie D. had suffered traumatizing physical and sexual abuse and lived in at least eight foster care (FC) placements. At six, when most children enter elementary school, Rosie entered a 3-month psychiatric hospitalization followed by back-to-back placements in residential facilities for exhibiting aggressive and self-injurious behaviors. Her foster parents sought intensive, home-based behavioral health services (HBHS) so Rosie could live safely at home, but their efforts to obtain such services failed. Instead, Rosie spent her formative years living in hospitals and institutions. In 2001, Rosie D. became the lead plaintiff in a class-action lawsuit against the state of Massachusetts on behalf of thousands of Medicaid-eligible children with serious emotional disturbance. In 2006, the court ruled Massachusetts in violation of federal Medicaid Early and Periodic Screening, Diagnostic and Treatment (EPSDT) provisions, which require that children receive medically necessary behavioral health services, including home- and community-based services. In response to this court decision, Massachusetts implemented a remediation plan increasing access to HBHS, with the hope that children like Rosie get a chance to grow up at home (Ponsor, 2006).

Today, nearly two decades after the landmark Rosie D. ruling, there are a growing number of children with behavioral health needs boarding in hospitals when not medically necessary (McEnany et al., 2020). Overwhelmed and under-supported caregivers, unable to access needed care for their child, are presenting to emergency rooms in crisis (McEnany et al., 2020). Children in FC are being abandoned at hospitals by foster parents or group home staff no longer able or willing to care for them. Some biological parents, after exhausting other options, are voluntarily relinquishing custody to child welfare in order to access behavioral health care (Hill, 2017). Once in the hospital, these children can remain there for weeks or months beyond medical necessity as child welfare searches for discharge placements equipped to meet their needs (Seltzer et al., 2022). Meanwhile, they are deprived of schooling, community activities, and socialization. In response, numerous state-level class action lawsuits have been filed on behalf of children in FC to prevent inappropriately restrictive hospital stays and expand the accessibility of HBHS (Oppenheim et al., 2012).

Among Medicaid-enrolled children, children in FC have the highest rates of use and mean expense for behavioral health services, with more restrictive settings (i.e., inpatient, residential treatment) making up a significantly higher percentage of spending than HBHS (Pires et al., 2018). Overprescribing of psychotropic medications has also been well-documented for this group (Pires et al., 2018). Expanding access to HBHS has the potential to address these challenges. Randomized trials show home- and community-based alternatives to pediatric inpatient psychiatric care have similar or better clinical outcomes, often with higher family satisfaction and lower costs (Kwok et al., 2016). Availability of consistent home-based supports may improve recruitment and retention of therapeutic foster parents willing to care for children with complex behavioral needs at home and avoid ED, inpatient, and residential use. Additionally, broadening access to HBHS may prevent parents from reaching the point of crisis that leads to voluntary FC placement (Hill, 2017).

Although all children in FC are Medicaid-eligible and states are required under EPSDT provisions to cover any services necessary to treat a child’s mental health condition, states maintain flexibility in determining HBHS delivery and reimbursement policies. This flexibility translates into wide differences in HBHS use. Using available Medicaid Analytic eXtraxt data from 2010–2012, we conducted a secondary analysis of Medicaid claims from 28 states with high quality data and found striking variation in the share of children with mental health diagnoses utilizing HBHS (Figure). During the sample period, between 41 to 78% (median 60%) of children in FC had an outpatient claim with a primary mental health diagnosis (ICD-9 codes for “Mental Disorders”, 290–319). Among those with a mental health diagnosis, less than half (0–43%, median 25%) accessed HBHS at least once (Figure). Examples of common HBHS accessed by children in FC included diagnostic services, individual and family therapy, and substance use disorder treatment. Across states, there is disconnect between the prevalence of mental health needs and access to HBHS (Fig. 1). For example, Oklahoma and Alabama have similar proportion of children in FC with a mental health condition (73% and 71%, respectively), but those living in Oklahoma were 10 times more likely to use HBHS (41% vs. 4%). While older data may not reflect current use, state variation in access to HBHS is likely driven by and persists due to state-specific Medicaid policies.

**Fig. 1:**
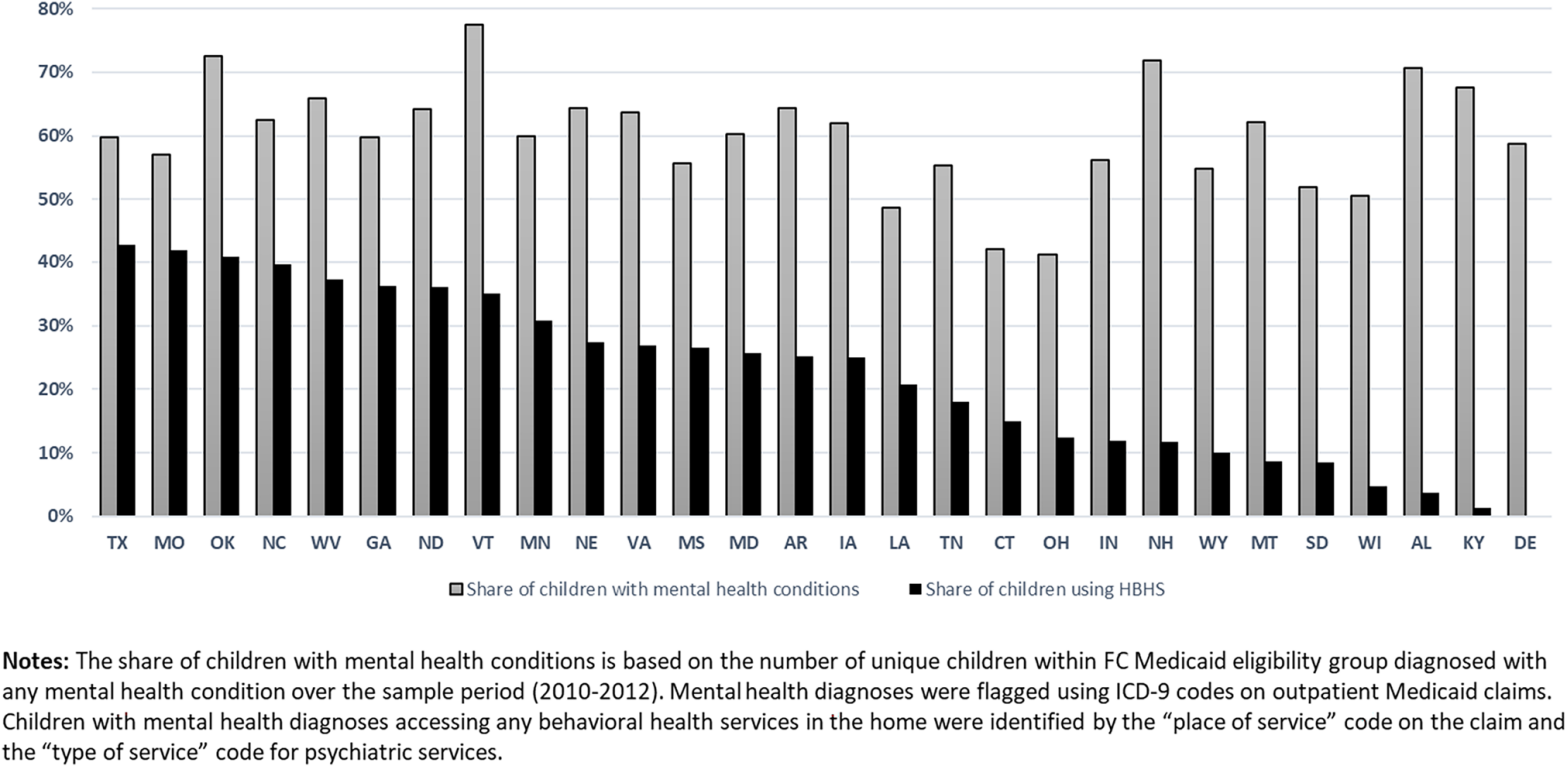
State Variation in Mental Health Prevalence and HBHS Use Among Children in FC. **a** Share of unique children within FC Medicaid eligibility group diagnosed with any mental health condition over the sample period (2010–2012). Mental health diagnoses were flagged using ICD-9 codes on outpatient Medicaid claims. **b** Share of children with mental health diagnoses accessing any behavioral health services in the home, defined by the “place of service” code on the claim and the “type of service” code for psychiatric services

Multiple states, including Iowa, Maryland, North Carolina, West Virginia, and most recently Georgia are facing ongoing lawsuits aiming to increase home- and community-based behavioral health services and prevent children in FC from boarding in hospitals. It is a critical opportunity for health policy and health services researchers to contribute to the discussion. We propose the following:

1. **Develop and disseminate a compendium of HBHS coverage**
  Health policy researchers should compile a clear, updated compendium that compares Medicaid coverage and reimbursement policies for HBHS by state. This compendium can be used a data source to highlight state-level differences in policies and identify gaps in existing coverage. The compendium could also serve as a resource for mental health providers and child welfare workers when determining which behavioral health services patients are eligible to receive and making referrals.
2. **Collect and analyze data on HBHS needs, use, and outcomes**
  There is a need to advocate for greater transparency regarding the number of children, both in and out of FC, boarding in hospitals and institutions due to behavioral health needs that could be managed in home-based settings if resources were available. While media reports from around the country highlight this problem, to our knowledge there are no state-level data publicly available nor any academic or policy publications with current statistics.
  Simultaneously, determining how many children are currently accessing HBHS and tracking child and health system outcomes could demonstrate which children benefit most from different HBHS models and estimate what services are most effective. Longitudinal child-level data linked across Medicaid, child welfare, behavioral health agencies, and other child serving agencies (i.e., education, juvenile justice)—complemented by a compendium of coverage—would allow researchers to use econometric and statistical analyses (such as quasi-natural experiments) to shed light on these questions. Application of this research could have a much broader reach than just state Medicaid coverage policies. For example, researchers could answer questions such as whether access to HBHS prevents initial entry to FC or impacts placement stability and permanency (e.g. reunification with biological parents, adoption) for children already in FC.
3. **Identify and systemically address specific barriers to accessing HBHS**
  Quantitative and qualitative research could reveal reasons behind observed state-level disparities in HBHS use and identify obstacles such as provider reimbursement rates, workforce challenges, provider and patient awareness, family preferences, and home safety concerns. Specific attention should be given to understanding barriers that uniquely impact access for children in FC, such as placement type (e.g., kinship or nonrelative foster home, group home) and time in care. In seeking solutions to the growing pediatric mental health crisis, particularly for children in FC, home-based care must be part of the conversation. The disconnect between prevalence of mental health needs and access to HBHS suggests widely varying Medicaid policies. By highlighting the potential of HBHS and pursuing needed reforms, we can better integrate home-based treatment options into the pediatric behavioral health care continuum. Driving efforts for data collection and transparency, advocating for expanded HBHS coverage, and posing critical empirical research questions about HBHS use and outcomes will ensure children in FC in every state receive the behavioral health care they need while living in the home they deserve.

## Abbreviations

FC: Foster care
HBHS: Home-based behavioral health services
