# Correction to: Expanding Access to HomeBased Behavioral Health Services for Children in Foster Care

**DOI:** 10.1007/s10488-024-01386-y

**Published:** 2024-05-25

**Authors:** Anna Chorniy, Michelle A. Mofa, Rebecca R. Seltzer

**Affiliations:** 1 Department of Medical Social Sciences, Feinberg School of Medicine, Northwestern University, Rubloff Building, 420 E. Superior St., Chicago, IL 60611 USA; 2 Buehler Center for Health Policy and Economics, Feinberg School of Medicine, Northwestern University, Chicago, IL USA; 3 Johns Hopkins School of Medicine, Baltimore, MD USA; 4 Johns Hopkins Bloomberg School of Public Health, Baltimore, MD USA

**Correction to: Administration and Policy in Mental Health and Mental Health Services Research** 10.1007/s10488-024-01357-3

The article “ **Expanding Access to HomeBased Behavioral Health Services for Children in Foster Care**”, written by **Anna Chorniy, Michelle A. Mofa, Rebecca R. Seltzer** was originally published electronically on the publisher’s internet portal on 7 February 2024 without open access. With the author(s)’ decision to opt for Open Choice the copyright of the article changed on 15 May 2024 to © The Author(s) 2024 and the article is forthwith distributed under a Creative Commons Attribution 4.0 International License, which permits use, sharing, adaptation, distribution and reproduction in any medium or format, as long as you give appropriate credit to the original author(s) and the source, provide a link to the Creative Commons licence, and indicate if changes were made. The images or other third party material in this article are included in the article’s Creative Commons licence, unless indicated otherwise in a credit line to the material. If material is not included in the article’s Creative Commons licence and your intended use is not permitted by statutory regulation or exceeds the permitted use, you will need to obtain permission directly from the copyright holder. To view a copy of this licence, visit http://creativecommons.org/licenses/by/4.0.

